# The unusual properties of lactoferrin during its nascent phase

**DOI:** 10.1038/s41598-023-41064-x

**Published:** 2023-08-29

**Authors:** Sara Notari, Giorgia Gambardella, Federica Vincenzoni, Claudia Desiderio, Massimo Castagnola, Alessio Bocedi, Giorgio Ricci

**Affiliations:** 1 Dipartimento di Scienze e Tecnologie Chimiche, Università di Roma “Tor Vergata”, Rome, Italy; 2 Dipartimento di Scienze biotecnologiche di Base, cliniche intensivologiche e perioperatorie, Università Cattolica del Sacro Cuore, Rome, Italy; 3 Fondazione Policlinico Universitario A. Gemelli IRCCS, Rome, Italy; 4 Istituto di Scienze e Tecnologie Chimiche “Giulio Natta”, Consiglio Nazionale delle Ricerche, Rome, Italy; 5 Laboratorio di Proteomica, Centro Europeo di Ricerca sul Cervello, IRCCS Fondazione Santa Lucia, Rome, Italy

**Keywords:** Protein folding, Protein folding, Proteins

## Abstract

Lactoferrin, a multifunctional iron-binding protein containing 16 disulfides, is actively studied for its antibacterial and anti-carcinogenic properties. However, scarce information is nowadays available about its oxidative folding starting from the reduced and unfolded status. This study discovers unusual properties when this protein is examined in its reduced molten globule-like conformation. Using kinetic, CD and fluorescence analyses together with mass spectrometry, we found that a few cysteines display astonishing hyper-reactivity toward different thiol reagents. In details, four cysteines (i.e. 668, 64, 512 and 424) display thousands of times higher reactivity toward GSSG but normal against other natural disulfides. The formation of these four mixed-disulfides with glutathione probably represents the first step of its folding in vivo. A widespread low p*K*_a_ decreases the reactivity of other 14 cysteines toward GSSG limiting their involvement in the early phase of the oxidative folding. The origin of this hyper-reactivity was due to transient lactoferrin-GSSG complex, as supported by fluorescence experiments. Lactoferrin represents another disulfide containing protein in addition to albumin, lysozyme, ribonuclease, chymotrypsinogen, and trypsinogen which shows cysteines with an extraordinary and specific hyper-reactivity toward GSSG confirming the discovery of a fascinating new feature of proteins in their nascent phase.

## Introduction

Lactoferrin (LF) is a member of transferrin family polypeptides, ables to regulate the level of free iron in mammals’ fluids. Due to its ability to bind iron with high affinity and to retain it at low pH, this protein displays antioxidant and bacteriostatic properties^1,2^. Other beneficial effects of LF have been described, including antiviral, anti-inflammatory and anti-carcinogenic properties^3^. The human LF is composed of a single polypeptide chain having 691 amino acids with a molecular mass of 77,250 Daltons. This protein contains 16 disulfides and its structure is able to bind two iron (*K* = 10^22^ M) and two carbonate ions^4^. Despite a lot of studies have been made detailing the tridimensional structure and many biological functions of LF, no information is available about its oxidative folding. The presence of many cysteines, all devoted to form disulfides, makes this step of particular interest for biochemists as it is reasonable the existence of mechanisms to avoid or repair the formation of incorrect disulfides. The present study discovers unusual properties of LF in its reduced molten globule-like structure which approaches to the transient architecture of this protein in its nascent phase. As observed in albumin^5^, another protein with many disulfides, as well as in lysozyme^6^, ribonuclease^7^, trypsinogen^8^ and chymotrypsinogen^9^ all having less disulfides, we found in LF a few structural cysteines, all devoted to form disulfides, displaying extraordinary and specific reactivity toward oxidized glutathione (GSSG). This finding suggests that these residues give mixed disulfide with GSH in the early phase of its oxidative folding. The glutathionylated residues become activated in their interaction with other protein cysteines resulting the privileged candidates to drive a hierarchical disulfide bond formation. This property, if limited to one or a few cysteines may be a strategy to avoid improper disulfide and, possibly, to avoid deleterious aggregations, as observed for lysozyme^6^. This paper explores the origin of this extraordinary reactivity and the identification of the involved cysteines, using circular dichroism (CD), fluorescence spectroscopy, kinetic and tandem mass spectrometry analyses. LF is involved in some serious pathologies^10–14^. For example, “corneal amyloidosis” is associated to the deposit of protein amyloid insoluble fibers formed by LF, not known if formed during the nascent phase or later^15^, but no specific LF mutations have been found associated to this disease . This paper examines the possibility that the very fast glutathionylation of a few cysteines in reduced LF (rLF) may prevent such deleterious aggregation.

## Results and discussions

### The molten globule status of rLF

**Figure 1:**
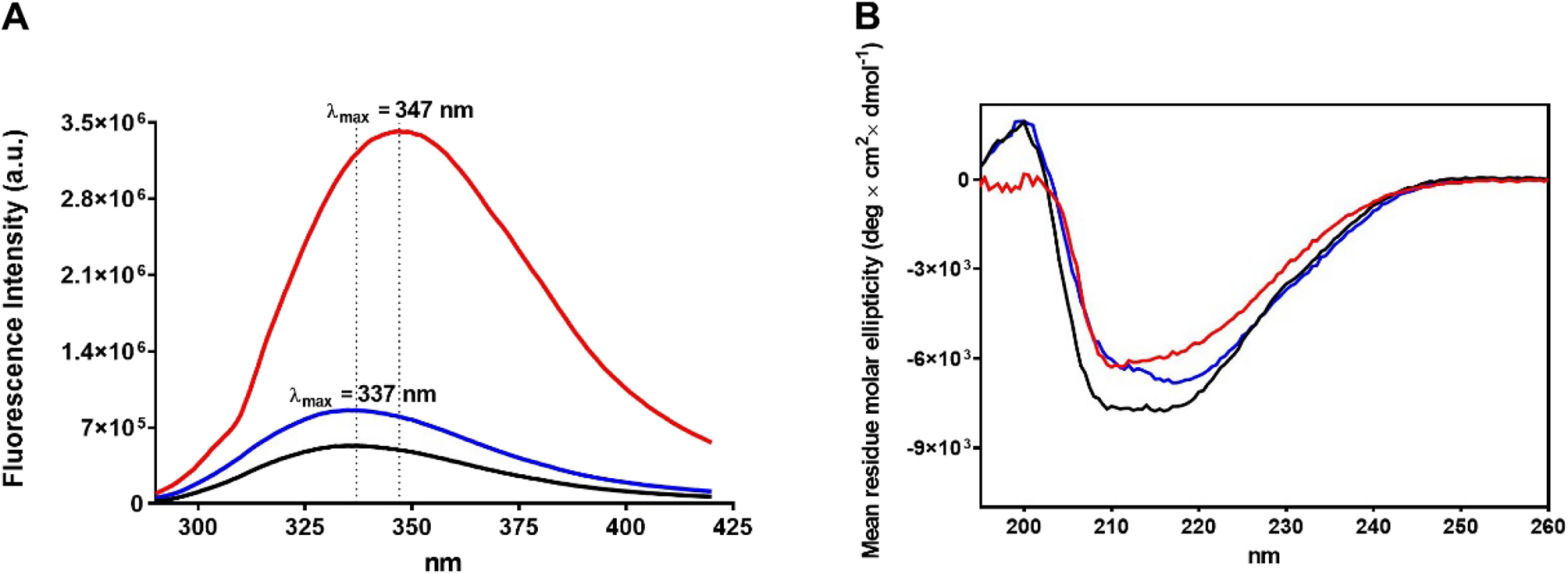
Circular dichroism and fluorescence spectra. (**A**) Intrinsic fluorescence spectra of native LF (1.1 μM, black line), native LF_p_ (1.1 μM, blue line), and rLF (1.1 μM, red line) in 0.2 M urea. (**B**) CD spectra of native LF (0.6 μM, black line), native LF_p_ (1.1 μM, blue line), and rLF (0.6 μM, red line) in 0.2 M urea. Both spectra were acquired in 0.1 M sodium acetate buffer pH 5.0 at 25 °C.

The "molten globule" represents a partially-folded protein status which can be formed under slightly denaturing conditions such as low pH, mild denaturant, or high temperature. It is considered an intermediate structure during the folding pathway of neo-synthetized proteins. Molten globules generally have some native-like secondary structure but a dynamic tertiary structure^16,17^. In the case of LF, its molten globule-like status was obtained after extensive reduction and denaturation with 25 mM dithiothreitol (DTT) in 8 M urea as described in Materials and methods. After removal of DTT, all the 32 cysteines were titratable with 2,2'-dithiobis(5-nitropyridine) (DTNP). When the reduced protein was kept in 0.2 M urea the resulting structure can be considered as a molten globule-like conformation. The fluorescence spectrum of this reduced protein is reported in Fig. 1A where a remarkable red shift of the maximum of fluorescence emission from 337 to 347 nm can be observed. This evidence, together with a strong enhancement of the fluorescence intensity (Fig. 1A), suggests that, in the molten globule-like structure, tryptophans and tyrosines are much more exposed to the solvent compared to the native protein. An additional contribution to this phenomenon can be also due to the breaking of the disulfides which are known to quench the fluorescence emission of tryptophan and tyrosines if located nearby^18,19^. The partially iron saturated lactoferrin (LF_p_) shows a fluorescence property similar to the native one (Fig. 1A).

CD spectra better define the different composition of secondary structures in the reduced molten globule-like status when compared to the native protein. From the spectra reported in Fig. 1B, a quantitative analysis performed using the DichroWeb software (Table 1)^20^, indicated that, in the reduced molten globule-like structure, about 50% of the α-helix disappeared with a partial conversion into β-sheet conformations. More slight differences are present in the turn structures. Conversely, the secondary structures of the LFp are more similar to the rLF (Table 1).

**Table 1:** Secondary structure analysis of CD spectra of LF reported in Fig. 1B.

	Helix (%)	Strand (%)	Turn (%)	Unordered (%)
Native LF	18.5	25.6	19.7	36.1
Native LF_p_	9.2	31.5	22.6	36.7
Reduced LF	8.4	29.6	23.4	38.6

### Reaction of rLF toward DTNB and alkylating reagents

**Figure 2:**
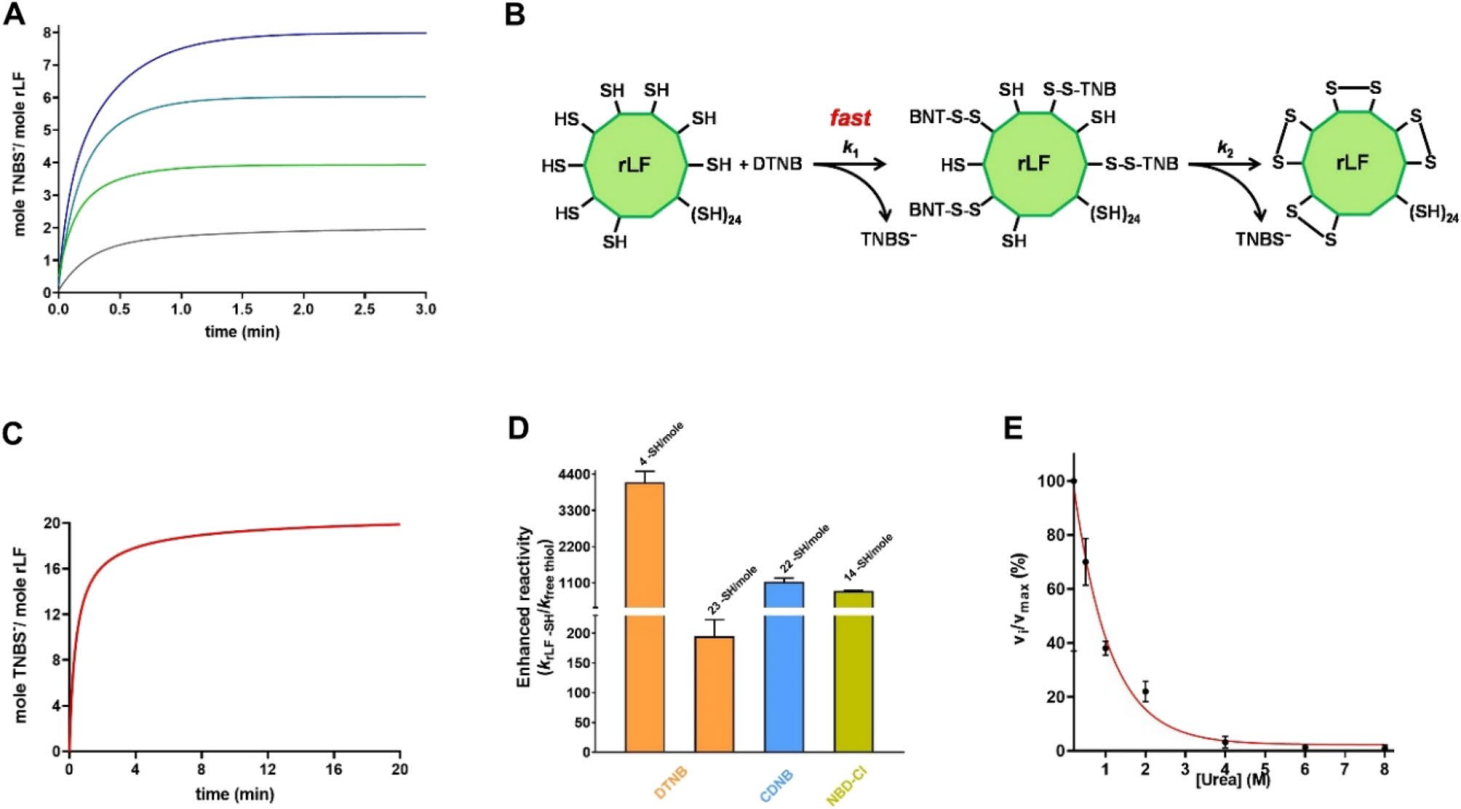
Reactivity of rLF cysteines toward DTNB and alkylating reagents. (**A**) TNBS¯ release after reaction of rLF (0.6 μM, 19.2 μM protein –SH) with sub-stoichiometric DTNB concentrations: 0.6 μM (ratio rLF:DTNB = 1:1, grey line); 1.2 μM (ratio rLF:DTNB = 1:2, green line); 1.8 μM (ratio rLF:DTNB = 1:3, dark cyan line); 2.4 μM (ratio rLF:DTNB = 1:4, blue line). The reactions were done in 0.2 M urea, 0.1 M sodium acetate buffer pH 5.0 at 25 °C. (**B**) Representative reaction scheme of rLF with sub-stoichiometric DTNB. (**C**) TNBS¯ release after reaction of rLF (0.6 μM, 19.2 μM protein –SH) with DTNB 9.6 μM (ratio rLF:DTNB = 1:16, red line). The reaction was done in 0.2 M urea, 0.1 M sodium acetate buffer pH 5.0 at 25 °C. (**D**) Second order kinetic constants of cysteines (*k*_rLF SH_) normalized to the rate constants for the free GSH (*k*_free thiol_). The number of protein cysteine per mole with a given reactivity is reported on the top of each column. Error bars represent the propagation of uncertainties for the quotients^21^. (**E**) Inhibition of the reactivity of cysteines in rLF (0.6 μM) toward DTNB (50 μM) by variable urea concentrations at pH 5.0 at 25 °C. The error bars represent the SD from three independent experiments.

The reactivity of the LF cysteines toward a few alkylating reagents and organic disulfides like 5,5’-dithiobis-(2-nitrobenzoic acid) (DTNB) was measured at pH 5.0, a pH value necessary to avoid the aggregation of the rLF, a phenomenon occurring at higher pHs. DTNB is a well known thiol reagent also used to titrate soluble thiols. In our case we observed that only 27 cysteines out of the 32 residues react with an excess of DTNB within 60 min (not shown). A few reduced cysteines are likely buried and masked by the structure of the molten globule, which displays consistent residual secondary structures compared to the native protein, as shown above by CD analysis. In any case interesting observations arise from this interaction. By incubating a stoichiometric amount of DTNB with rLF (rLF:DTNB = 1:1 i.e. DTNB:protein cysteines = 1:32), two equivalents of TNBS^−^ were released suggesting that the mixed disulfide protein-S–S-TNB rapidly evolved to a symmetric protein disulfide releasing the second ion TNBS^−^ (Fig. 2A). A similar phenomenon was observed up to an rLF:DTNB 1:4 ratio with the corresponding release of 8 TNBS^−^. These four cysteines are the only residues that give a very fast intramolecular disulfide (Fig. 2B). In fact, by reacting an amount of DTNB corresponding to 50% of the cysteines of LF (rLF:DTNB = 1:16 i.e. DTNB:protein cysteines = 16:32), 20 TNBS^−^ were released indicating that only these four disulfides are formed (Fig. 2C). Kinetic analyses concerning the first four fast reacting cysteines gave an average rate constant 4150 times higher than that of a normal protein cysteine. Other 23 cysteines are less reactive but always 195 time higher (Table 2 and Fig. 2D). All these hyper-reactivities were completely lost in a denaturation medium as shown by the effect of increasing urea concentrations (Fig. 2E). As DTNB has no biological interest, we did not identify the hyper-reactive cysteines toward this reagent.

**Table 2:** Reactivity of cysteines of fully reduced LF toward DTNB and alkylating reagents. Data are Mean ± SD.

Reagents	Free GSH	Reduced LFs			
	*k* (M^−1^ s^−1^)	*k* (M^−1^ s^−1^) (–SH/mol LF)	Enhanced reactivity	*k* (M^−1^ s^−1^) (–SH/mol LF)	Enhanced reactivity
DTNB	20	83,040 ± 6750 (4 Cysteines)	4150 ± 340	3910 ± 550 (23 Cysteines)	195 ± 30
CDNB	0.00028	0.32 ± 0.03 (22 Cysteines)	1130 ± 110	–	–
NBD-Cl	0.032	27 ± 1 (14 Cysteines)	840 ± 30	–	–

Hyper-reactivity was also found for 1-chloro-2,4-dinitrobenzene (CDNB) an alkylating reagent which reacted with 22 protein cysteines following an apparent monophasic trend (not shown). The rate was 1130 times higher than a normal protein cysteine. A similar hyper-reactivity (840 times higher) was also observed for 14 cysteines of rLF in their reaction with 4-chloro-7-nitrobenzofurazane (NBD-Cl), a second alkylating reagent (Table 2 and Fig. 2D).

### Reaction with GSSG and other natural disulfides

**Figure 3:**
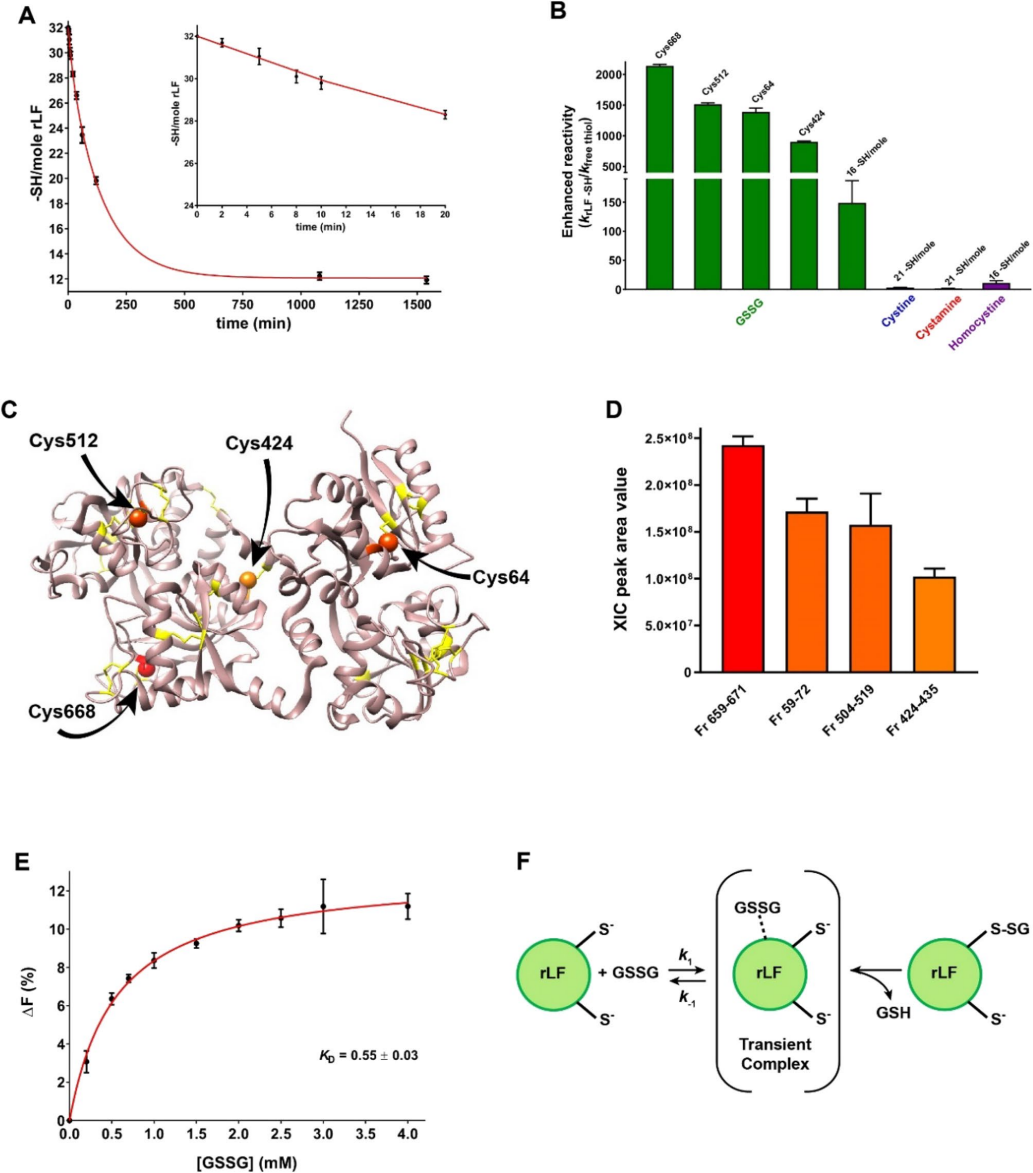
Reaction of rLF toward GSSG and natural disulfides. (**A**) Disappearance of rLF cysteines (1 µM, 32 µM protein –SH) during the reaction with 1 mM GSSG in 0.2 M urea, 10 mM sodium acetate buffer pH 5.0, at 25 °C. The 32 –SH/mole of rLF were determined with DTNP. The error bars represent the SD from three independent experiments. The first phase of reaction (up to 20 min) when only a few cysteines reacted with GSSG is reported in the Inset. (**B**) Second order kinetic constants of cysteines (*k*_rLF SH_) normalized to the rate constants for the free GSH or, only for GSSG, normalized to the constant calculated for an unperturbed protein cysteine (*k*_free thiol_). The number of protein cysteine per mole with a given reactivity is reported on the top of each column. Error bars represent the propagation of uncertainties for the quotients^21^. (**C**) 3D structure of native rLF (PDB id: 1LFG)^25^ is depicted in pink ribbons with cysteines in yellow sticks. The four most reactive cysteines toward GSSG identified by MS analysis are displayed in four different orange palettes (spheres). The amino acid number in black derived from X-ray structure in PDB database. The 3D representation is obtained by UCSF chimera software^26^. (**D**) Peak area values of the four glutathionylated tryptic peptide fragments after reaction with GSSG (see Materials and methods for details) as revealed by the eXtracted Ion Current procedure. No other modified fragments were detected. Error bars represent the SD from three analytical replicate LC–MS analyses. (**E**) Quenching of the intrinsic fluorescence of rLF (1.1 µM) after addition of variable GSSG concentrations (from 0.2 to 4 mM) in 0.2 M urea, 10 mM sodium acetate buffer pH 5.0 at 25 °C. Each point was subtracted by the fluorescence of NATA with GSSG under the same conditions. Measurements were made immediately after mixing to avoid any glutathionylation interference. The error bars represent the SD from three independent experiments. (**F**) Schematic representation of the reaction of rLF with GSSG.

Glutathione is the natural tripeptide actively involved in the maintenance of the reduced status of the cell. In the cytosol and in various cell compartments concentration of the reduced form (GSH) is about 100 fold higher than the oxidized form (GSSG). Glutathione reductase, an NADPH dependent enzyme, is intended to produce a continuous reduction of GSSG which can be formed by endogenous or exogenous oxidizing compounds like peroxides^22^. The GSH/GSSG ratio is singularly different in the endoplasmic reticulum (ER), where the GSSG concentration reaches 1/5 the one of GSH, i.e., 0.4 mM and 2 mM, respectively^23^. Other studies reported that, in ER, GSSG level may reach even 2 mM^24^. This unusual GSSG concentration is sound because most of the oxidative folding pathways of proteins occur in ER. In the present study we observed that GSSG reacts with 20 protein cysteines. Due to the large number of residues reacting with GSSG, the analysis of the –SH disappearance shown in Fig. 3A, does not allow to discriminate the presence of some cysteines with particular hyper-reactivity toward GSSG. By assuming that GSSG reacts with similar rates with all the 20 residues involved, an average kinetic constant of 0.15 M^−1^ s^−1^ can be estimated, corresponding to an enhanced reactivity of about 190 when compared to the reactivity of an unperturbed protein cysteine. This finding is of particular interest as other natural disulfides like cystine, homocystine and cystamine, all able to react with many protein cysteines of rLF, show null or very small enhanced reactivity (Table 3 and Fig. 3B).

**Table 3:** Reactivity of cysteines of fully reduced LFs toward natural disulfides. ^a^ Theoretical value for unperturbed protein cysteine (p*K*_a_ = 9.1); Data are Mean ± SD.

Reagents	Unperturbed protein Cys	Free GSH	Reduced LFs			
	*k* (M^−1^ s^−1^)	*k* (M^−1^ s^−1^)	From kinetics		From kinetics and mass spectrometry data	
			*k* (M^−1^ s^−1^) (–SH/mol LFs)	Enhanced reactivity	*k* (M^−1^ s^−1^) (–SH/mol LFs)	Enhanced reactivity
GSSG	0.0008^a^		0.15 ± 0.02 (20 Cysteines)	190 ± 25	1.71 ± 0.02 (Cys668)	2140 ± 25
					1.21 ± 0.02 (Cys512)	1510 ± 25
					1.11 ± 0.05 (Cys64)	1390 ± 60
					0.72 ± 0.01 (Cys424)	900 ± 13
					0.12 ± 0.03 (16)	150 ± 40
Cystine		0.048	0.17 ± 0.02 (21 Cysteines)	3.5 ± 0.4	–	–
Cystamine		0.22	0.052 ± 0.005 (21 Cysteines)	0.24 ± 0.02	–	–
Homocystine		0.0024	0.03 ± 0.01 (16 Cysteines)	11 ± 4	–	–

Motivated by these results, we performed a mass spectrometry analysis of the LF after short incubation times with GSSG in the attempt to obtain more information on the specific cysteines involved in the GSSG interaction, and on the possible presence of one or more cysteines residues with a specifically high hyper-reactivity.

After 2, 5 and 10 min of incubation of rLF with GSSG, the reaction was stopped by addition of bromopyruvate (BrP), a reagent able to alkylate all protein cysteines within a few seconds. The tryptic digestion of these samples, analyzed by mass spectrometry, revealed that, at any time, only four residues were partially glutathionylated, i.e. Cys64, Cys424, Cys512 and Cys668 (Fig. 3C). A relative quantitative analysis of the peptide fragments, based on the peak area values of the eXtracted Ion Current (XIC) plots, observed after only 2 min of incubation, (Fig. 3D) allowed us to evaluate the corresponding kinetic constants reported in Table 3. It appears that C668 is the most active residue, showing a reactivity 2140 times higher than an unperturbed protein cysteine, while Cys64 and Cys512 have enhanced reactivities of 1510 and 1390, respectively. Finally, the fourth reactive Cys424 shows about 900 times increased reactivity.

### Fluorescence quenching due to GSSG interaction

In an attempt to have some insights about the origin of the extraordinary hyper-reactivity toward GSSG exhibited by four cysteines, we performed florescence experiments assaying the intrinsic fluorescence emission of the tryptophans and tyrosines after mixing rLF with variable amount of GSSG. Fluorescence perturbations were monitored within a few seconds from any addition of GSSG, before the formation of any mixed disulfides between GSSG and protein cysteines. Results shown in Fig. 3E depict a fluorescence quenching due to GSSG binding with a monophasic hyperbolic behavior. These results point for the formation of one or more transient rLF-GSSG complexes with an apparent *K*_D_ of 0.55 mM. We believe that the quenching observed can be referred to a single binding site for GSSG able to give a productive interaction with one protein cysteine but even with the other three reactive cysteines in the very flexible molten globule-like structure (Fig. 3F). Alternatively, other binding sites for GSSG can be present, but the reversible link with GSSG does not produce any fluorescence perturbation. Less probable is the existence of four binding sites for GSSG with similar affinity.

### An unusual low p***K***_a_ characterized many reactive cysteines in the rLF

**Figure 4:**
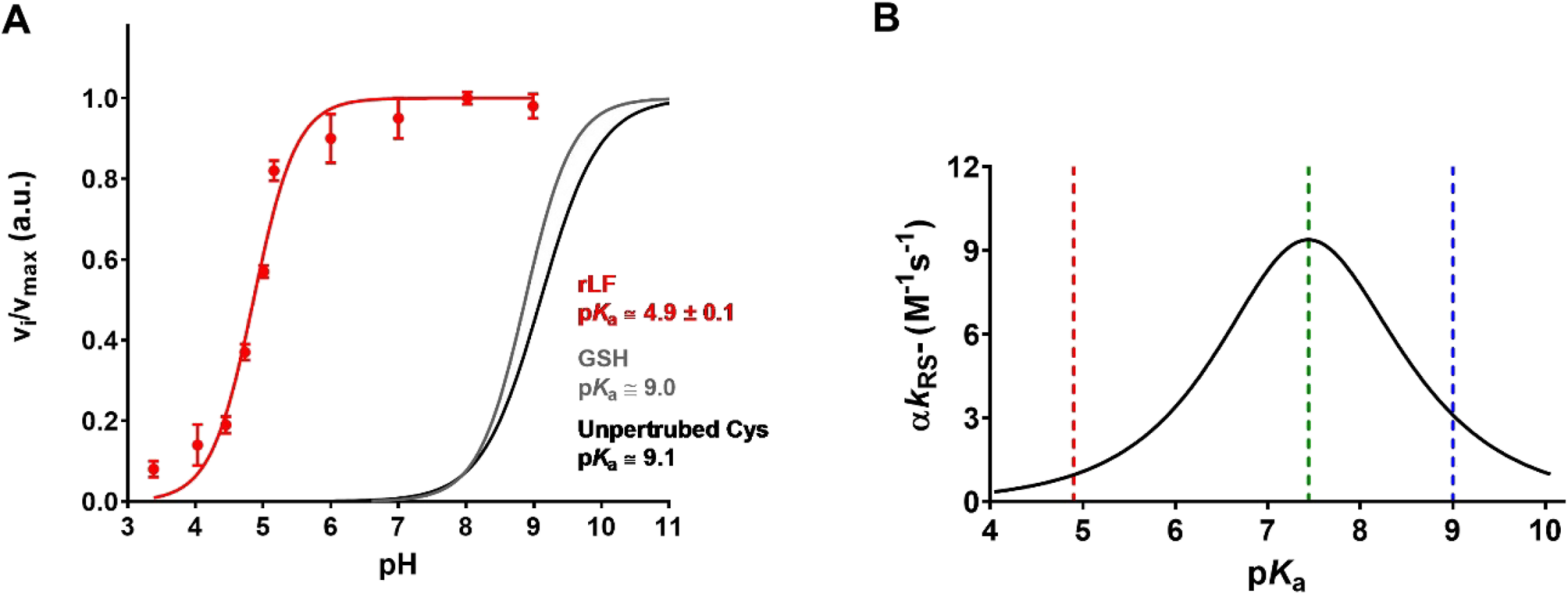
Average p*K*_a_ determination. (**A**) The rLF (0.6 μM) was reacted with NBD-Cl (0.1 mM) in 0.2 M urea at variable pH values, 25 °C (red line). The average p*K*_a_ estimated was 4.9 ± 0.1. The error bars represent the SD from three independent experiments. As a control the p*K*_a_ value of GSH (p*K*_a_ = 9.0, grey line) is reported according to Ref. 9. The theoretical curve of an unperturbed protein cysteine (p*K*_a_ = 9.1, black line) is also reported. (**B**) Dependence of the second order kinetic constants (α*k*_RS-_) on p*K*_a_ for the reaction of several thiols with disulfides at pH 7.4. The p*K*_a_ values of 4.9, 7.4, and 9.0 are marked with red, green, and blue dotted lines, respectively. Figure modified from Ref. 29.

A further very unusual property of many reactive cysteines of the molten globule-like structure of rLF is their very acidic properties. The reactivity toward NBD-Cl at various pHs (Fig. 4A) defines a low average p*K*_a_ of 4.9, about four unit lower than that of a unperturbed protein sulfhydryl (p*K*_a_ = 9.1)^27^ (an older study reports a lower p*K*_a_ ~ 8.5)^28^. A usual but incorrect opinion is that a low p*K*_a_ always makes more reactive the sulfhydryl group of a protein cysteine increasing the amount of the thiolate, the only reactive form of the sulfhydryl group. However, a relevant decrease of nucleophilicity parallels an increased acidity so, in a reaction with disulfides occurring at pH 7.4, the reaction rate of a thiol with p*K*_a_ = 4.9 is lower than that of a normal sulfhydryl with p*K*_a_ = 8.5–9.0 (Fig. 4B). Thus, the very unusual acidity of many cysteines in rLF may be explained by the need to decrease their reactivity avoiding the formation of incorrect disulfides.

### Influence of the fast glutathionylation on the aggregation of rLF

The molten globule-like conformation of rLF is stable only at pH values ≤ 5.0. At physiological pHs this protein undergoes insoluble aggregates. We observed a similar phenomenon in other reduced proteins like lysozyme, chymotrypsinogen and trypsinogen^6,8,9^. In the case of lysozyme, the rapid glutathionylation of its hyper-reactive cysteine (Cys94) lowered its irreversible aggregation. In the case of rLF either the pre-incubation with GSSG at pH 5.0 and the subsequent increased pH to 7.4, or the direct incubation with GSSG at pH 7.4, do not yield any appreciable quenching of aggregation.

**Figure 5:**
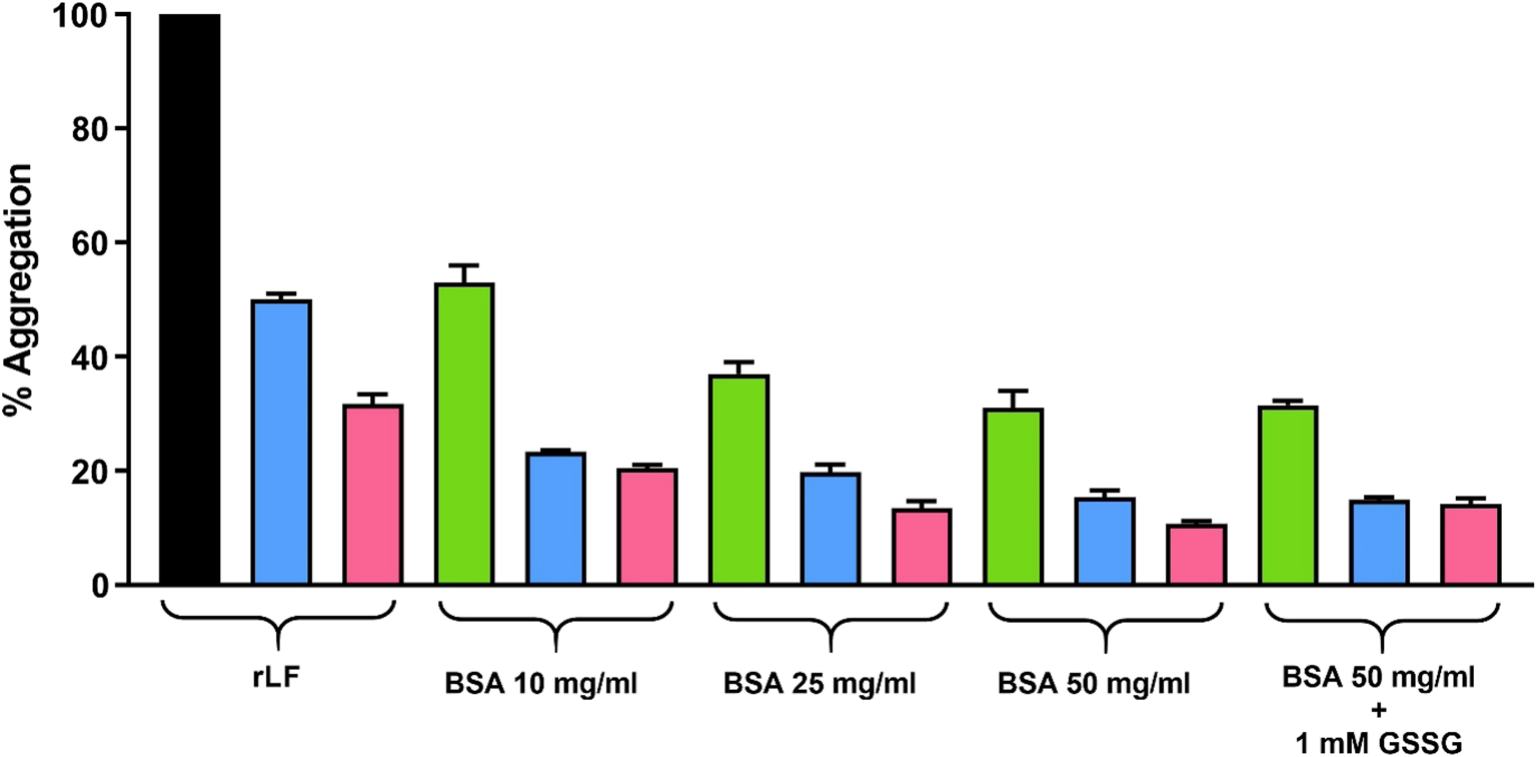
Aggregation of rLF. Aggregation of rLF in absence, or in presence of different BSA concentrations (from 10 mg/ml to 50 mg/ml) and in presence of BSA 50 mg/ml and GSSG 1 mM. The different color represents the different protein concentration: rLF 0.6 μM (green columns); rLF 0.3 μM (blue columns) and rLF 0.2 μM (pink columns). All the percentage values are referred to the aggregation of rLF 0.6 μM alone (black column). All the experiments were performed in 0.2 M urea, 10 mM sodium phosphate buffer pH 7.4, at 25 °C. The error bars represent the SD from three independent experiments.

However, if the rLF is incubated at pH 7.4 in the presence of increasing amount of albumin, the aggregation was lowered, reaching 90% at 50 mg/ml albumin concentration (Fig. 5). Considering that albumin concentration in the ER is about 100 mg/ml^30^ and about 300 mg/ml in the cytosol, all these levels are probably able to limit any deleterious aggregation of rLF in vivo. Thus, the presence of the four hyper-reactive cysteines toward GSSG is probably only necessary to dictate a proper temporal sequence of disulfide formation.

## Conclusions

The present study discovers novel properties of a few cysteines of LF in its molten globule-like conformation, a particular structure which is reminiscent of its structure during the nascent phase. It is general opinion that the reactivity of the sulfhydryl group of protein cysteines is mainly determined by its acidity but, in a recent study, we found that this parameter cannot increase the reactivity more than three/four times and that a remarkable acidity depresses the rate of the reaction toward disulfides or alkylating reagents (see Fig. 4B)^29^. Other, more important factors must be considered that may speed the reaction hundred and even thousand times, i.e., hydrophobic or ionic interactions, which make sulfhydryl and reagents able to react productively^29^. These speeding factors must be present in rLF to justify the extraordinary hyper-reactivity of four cysteines of rLF toward DTNB (more than 4000 times over the one of a normal protein cysteine) and the thousand times increased reactivity found for many cysteines toward two hydrophobic reagents like CDNB and NBD-Cl (22 and 14 cysteine, respectively). All these findings depict curious and also interesting properties but they have a limited or null physiological relevance due to the absence of these compounds into the cell. Conversely, the kinetic properties toward GSSG of four selected cysteines, identified by mass spectrometry, namely, Cys64, Cys424, Cys512 and Cys668, discloses a new feature of the molten globule-like status which may enlighten the early step of LF oxidative folding. In fact, GSSG is present at an unusual high concentration in the ER (in the range 0.4–2 mM)^23,24,31^ and becomes able to produce a fast glutathionylation of the hyper-reactive cysteines of rLF as well as of other proteins. In this context, a recent study identified in the ribosomal exit tunnel some glutathionylated cysteines in the nascent bovine γB-crystallin^32,33^. The presence of these mixed disulfides is a clear indication of a role of GSSG in the oxidative folding of other proteins and of the need of relevant hyper-reactivity for the involved cysteines. This evidence seems to contradict the current opinion that GSSG is not directly involved in the oxidative folding^34^. The origin of the hyper-reactivity toward GSSG in rLF may be explained assuming the occurrence of one or more rLF-GSSG transient complexes which have been signaled by fluorescence experiments (Fig. 3E). In agreement with this scenario is also the absolute specificity toward GSSG given that a normal kinetic behavior have been found with other natural disulfides like cystine, cystamine and homocystine (Table 3). We note also that the calculated *K*_D_ (0.55 mM) enters in the range of GSSG level found in the ER. A sure finality for the observed hyper-reactivity in rLF cannot be deduced and can be only hypothesized. One reasonable utility may be that the hyper-reactive cysteines becomes the first residues able to form disulfides, avoiding the formation of non-natural disulfides, an event highly probable in the presence of 32 cysteines having comparable reactivity. Noteworthy is also the particular very low p*K*_a_ values of many cysteines, a property that slows down their reactivity toward GSSG, further enhancing the propensity of the four hyper-reactive cysteines to supply the natural disulfides first. A similar sequential events of disulfide formation was also observed in albumin which also displayed a few cysteines with different hyper-reactivity^5^. The possibility that a very fast glutathionylation may limit the deleterious aggregation, as found in lysozyme, was not confirmed for rLF. The presence of surrounding proteins, in a concentration similar or lower to that found in the ER, should be sufficient to repress the aggregation when this protein is present in the nascent reduced form.

In conclusion, we are honored to offer to the international community of biochemists a novel and unknown property of the molten globule-like structure which may help to better detail the early step of the oxidative folding of other proteins having disulfides in their structure. Many opinions about the scarce or null presence of an “intelligent” structure in this transient status could be revised by considering the ability to bind productively GSSG in a transient complex which selectively catalyzes the very fast glutathionylation of selected cysteines. Albumin, lysozyme, ribonuclease, trypsinogen and chymotrypsinogen are the other disulfide containing proteins that have been found to have similar selected hyper-reactive cysteines toward GSSG^5–9^. Our aim will be to continue the discovery of other disulfide containing proteins showing similar hyper-reactive cysteines in their nascent structures. Thus, we appeal to all research groups who are studying proteins containing disulfides and with the availability of a few milligrams, to get in touch with our group to start collaboration on this issue.

## Materials and methods

### Chemicals and reagents

Human Lactoferrin (LF) recombinant, expressed in rice, Iron saturated, ≥ 90% (SDS-PAGE), Human Lactoferrin partially iron saturated (LF_p_ iron saturation ~ 0.07%) recombinant, expressed in rice, ≥ 90% (SDS-PAGE), dithiothreitol (DTT), urea, ethylendiamminotetraacetic acid (EDTA), 5,5′-dithiobis (2-nitrobenzoic acid) (DTNB), 1-chloro-2,4-dinitrobenzene (CDNB), 4-chloro-7-nitrobenzofurazane (NBD-Cl), N-acetyl-L-tryptophanamide (NATA), bromopyruvic acid (BrP), cystamine, cystine, homocystine, oxidized glutathione (GSSG), and ammonium bicarbonate (AMBIC) was from Sigma-Aldrich (St. Louis, MO, USA). Water and formic acid (FA), LC–MS grade, were from Merck (Darmstadt, Germany). Trypsin enzyme (Gold MS Grade) was from Promega (Madison, WI, USA). Acetonitrile (ACN) (LC–MS grade) was from Merck (Darmstadt, Germany).

### Protein reduction

The LF concentration was estimated in 0.1 M sodium acetate buffer at 280 nm (ε = 88,290 M^−1^ cm^−1^)^35^. The LF (8 mg) was solubilized in 8 M urea, 1 mM EDTA, 50 mM sodium phosphate buffer pH 7.4 (1 mL) and reduced with 25 mM DTT (LF:DTT = 1:260) for 60 min at 60 °C. The excess of DTT was removed by passing the reduced protein trough Sephadex G-25 column (1 X 20 cm) equilibrated with 8 M urea, 1 mM EDTA, 10 mM sodium phosphate buffer pH 7.4 (throughout the manuscript, the reduced lactoferrin after this process is indicated as rLF). The number of –SH/mole of the eluted protein was titrated with DTNB at pH 5.0 (ε_412_ = 11,800 M^−1^ cm^−1^ at pH 5.0 for TNBS^–^)^5^ in the presence of 2 M urea at 25 °C. Alternatively, all the 32 –SH/mole of the eluted protein were titrated with DTNP^36^ at pH 5.0 (ε_386_ = 14,860 M^−1^ cm^−1^ at pH 5.0 for 2-mercapto-5-nitropyridine). The native LF did not show free –SH groups as observed with DTNB.

### Fluorescence analysis

The fluorescence measurements of native LF (1.1 μM), native LF_p_ (1.1 μM), and rLF (1.1 μM) in 0.2 M urea were performed in 10 mM sodium acetate buffer pH 5.0. For the determination of the *K*_D_ for rLF-GSSG interaction the spectra were recorded with a protein concentration of 1.1 μM in 0.2 M urea, 10 mM sodium acetate buffer pH 5.0 in the presence of different GSSG concentrations (from 0.2 to 4 mM). The maximum intensities of each spectra (347 nm) were subtracted by the maximum intensities (361 nm) acquired for NATA in the same conditions. All the experiments described were performed on a Fluoromax-4 Horiba spectrofluometer using an asymmetric quartz cuvette (1 × 0.4 cm path length) at 25 °C. The setting panel were: slit 2–5 nm, excitation wavelength 280 nm, and emission spectra 290–420 nm (or 320–380 nm for the *K*_D_ determination).

### Circular dichroism spectroscopy

The CD spectra of native LF, LF_p_, and rLF in 0.2 M urea were performed with a protein concentration of 0.6 μM in 10 mM sodium acetate buffer pH 5.0 at 25 °C. The spectra were recorded in a quartz cuvette of 0.5 cm path length using a spectropolarimeter Jasco J-1500 (Easton, MD) and the following setting panel: slit 2 nm, sensibility 20 mdeg, resolution 0.5 nm, and range 195–260 nm. The analyses of CD spectra were performed using DichroWeb^20^.

### Reaction of rLF toward alkylating reagents and DTNB

The reactivity of rLF toward the alkylating reagents was established as follows. The rLF (0.6 μM) was reacted with NBD-Cl (0.1 mM) in 0.1 M sodium acetate buffer pH 5.0 and 0.2 M urea at 25 °C. The reaction was followed spectrophotometrically in continuous at 419 nm, where the Cys-NBD adduct absorbs (ε_419_ = 13,000 M^−1^ cm^−1^)^37^. The reactivity of rLF (0.6 μM) toward CDNB (0.5 mM) was performed in 10 mM sodium acetate buffer pH 5.0 and 0.2 M urea at 25 °C. The reaction was followed spectrophotometrically in continuous at 340 nm, where the Cys-DNB adduct absorbs (ε_340_ = 9600 M^−1^ cm^−1^)^5^. The reactions of rLF (0.6 μM) toward DTNB (16.3 μM) was evaluated in 0.1 M sodium acetate buffer pH 5.0 in the presence of 0.2 M urea at 25 °C, while, alternatively, toward DTNB 50 μM in 0.1 M sodium acetate buffer pH 5.0 and 2 M urea at 25 °C. For reactions of rLF (0.6 μM) with sub-stoichiometric amounts of DTNB, the evaluation was performed in 0.2 M urea, 0.1 M sodium acetate buffer pH 5.0 (25 °C) with DTNB 0.6 μM, 1.2 μM, 1.8 μM, and 9.6 μM for the ratio rLF:DTNB = 1:1, rLF:DTNB = 1:2, rLF:DTNB = 1:3, rLF:DTNB = 1:4, and ratio rLF:DTNB = 1:16, respectively. The reactions in 0.2 M urea was performed using the SFA-12 Rapid Kinetics Accessory (Hi-Tech Scientific, Bradford-on-Avon, UK). The reference reactions of the free cysteine with GSSG and the free GSH with all other reagents were reported in a previous study^9^.

### Effect of urea on the reactivity of rLF

The effect of urea concentration on the reactivity of rLF cysteines was assayed using DTNB as follows: 0.6 μM rLF was incubated with 50 μM DTNB in 0.1 M sodium acetate buffer, pH 5.0 (25 °C) containing variable concentrations of urea (from 0.2 M to 8 M). The reaction rate was measured spectrophotometrically at 412 nm using the rapid-mixing apparatus.

### Reaction of rLF toward GSSG and other natural disulfides

The reactions of rLF towards GSSG (1 mM), cystine (0.5 mM), cystamine (1 mM) and homocystine (1 mM) were conducted indirectly incubating 1 µM of the reduced protein with each disulfide. The reaction was carried out in 0.2 M urea, 10 mM sodium acetate buffer pH 5.0 at 25 °C. At different times, an aliquot of the reaction mixture was acidified with HCl 1.2 M (final pH ≈ 2.0) and the solution was subsequently centrifuged at 12,000 g for 10 min on Vivaspin-500 (10 kDa membrane cut-off) (Sartorius, Germany). The filtrate was brought to pH 8.0 with sodium phosphate buffer 1 M and the thiols content titrated with DTNB (ε_412_ = 14,100 M^−1^ cm^−1^ at pH 8.0 for TNBS^−^)^38^. The maximum incubation times were around 26 h for GSSG and homocystine, and around 23 h for cystine and cystamine.

### Preparation of rLF samples for mass-spectrometry analysis

The rLF (1.4 μM) was incubated with GSSG (1 mM) in 10 mM sodium acetate buffer pH 5.0 in the presence of 0.2 M urea at 25 °C. After 2, 5 and 10 min the reaction was stopped by adding 1 mM BrP that alkylates residual protein cysteines within 1–2 s. As a control, a solution containing rLFs (1.4 μM) was immediately alkylated with BrP 1 mM in 10 mM sodium acetate buffer pH 5.0 (25 °C).

### Filter-aided sample preparation protein digestion

Filter-aided sample preparation (FASP) centrifugal Microcon filtration devices (Millipore) with 10 kDa molecular mass cut-off filter membrane were used for rLF samples digestion by trypsin. A volume of each sample corresponding to 20 µg of total protein content was added of Ammonium Bicarbonate Buffer (AMBIC) to reach a final volume of 200 µL, then transferred to the filter device and centrifuged at 14,000 rpm for 15 min at thermostated temperature (+ 4 °C). The filter supernatant was then washed three times with AMBIC to remove urea and to ensure successful buffer exchange for trypsin digestion conditions. Sample digestion was carried out overnight at 37 °C adding to the filter 1:50 (w/w) trypsin enzyme solution 1 µg/µL in 50 mM AMBIC. Enzymatic digestion was stopped by addition of 1% FA (final concentration). The proteolytic peptides were collected by centrifugation, lyophilized, and dissolved in 0.1% FA water solution (v/v) for LC–MS analysis.

### Ultra high performance liquid chromatography-nanoESI mass spectrometry analysis (UHPLC-ESI–MS/MS)

UHPLC-ESI–MS/MS analyses were performed for each sample in triplicate on UltiMate 3000 RSLCnano System coupled to Orbitrap Elite MS detector with EASY-Spray nanoESI source (Thermo Fisher Scientific, Waltham, MA, USA) and equipped with Thermo Xcalibur 2.2 computer program (Thermo Fisher Scientific) for instrumental operation and data acquisition. Chromatographic separations were performed on PepMap C18 (2 µm particles, 100 Å pore size) EASY-Spray column 15 cm in length × 50 µm of internal diameter (ID) (Thermo Fisher Scientific) in coupling to Acclaim PepMap100 nano-trap cartridge (C18, 5 µm, 100 Å, 300 µm i.d. × 5 mm) (Thermo Fisher Scientific). Separation were performed at 40 °C in gradient elution using 0.1% FA as eluent A and an ACN/FA solution (99.9:0.1, v/v) as eluent B as following: (i) 5% B (7 min), (ii) from 5 to 35% B (113 min), (iii) from 35% B to 99% (2 min), (iv) 99% B (3 min), (v) from 99% to 1.6% B (2 min), (vi) 1.6% B (3 min), (vii) from 1.6% to 78% B (3 min), (viii) 78% B (3 min), (ix) from 78% to 1.6% B (3 min), (x) 1.6% B (3 min), (xi) from 1.6% to 78% B (3 min), (xii) 78% B (3 min), (xiii) from 78% B to 5% B (2 min), (xiv) 5% B (20 min). The mobile phase flow rate was 0.3 µL/min. The injection volume was 5 µL. The Orbitrap Elite instrument operated in positive ionization mode at a 60,000 full scan resolution in 350–2000 m/z acquisition range, performing MS/MS fragmentation by collision-induced dissociation (CID, 35% normalized collision energy) of the 20 most intense signals of each MS spectrum in Data-Dependent Scan (DDS) mode. The minimum signal was set to 500.0, the isolation width to 2 m/z and the default charge state to + 2. MS/MS spectra acquisition was performed at resolution of 60,000 and setting isolation width to 2 m*/z*.

### p***K***_a_ determination

The average p*K*_a_ of cysteines in the rLF (0.6 μM) was determined using NBD-Cl (0.1 mM) in 10 mM Britton-Robinson buffer (pH varying from 3.4 to 9.0) at 25 °C. The aggregation rate of rLF between pH 5.2 and pH 8.5 was subtracted to the reaction rate between rLF and NBD-Cl at the same pH value. The initial velocities obtained were normalized respect to the maximum velocity observed, then the p*K*_a_ value was calculated by a curve fitting analysis.

### Aggregation studies

The rLF aggregation process was evaluated at different protein concentrations (0.6 µM, 0.3 µM and 0.2 µM) in absence and in presence of variable amounts of BSA (10 mg/ml, 25 mg/ml and 50 mg/ml). The experiment with BSA 50 mg/ml was also performed in presence of 1 mM GSSG. All experiments were conducted in 0.2 M urea, 10 mM sodium phosphate buffer pH 7.4 at 25 °C, monitoring the process spectrophotometrically in continuous at 600 nm.

### Data analysis and graphical representation

LC–MS and MS/MS data were elaborated by the HPLC–MS apparatus management software (Xcalibur 2.0.7 SP1, Thermo Fisher Scientific), and by means of Proteome Discoverer 1.4 software (version 1.4.1.14, Thermo Fisher Scientific) for protein characterization based on SEQUEST HT cluster as search engine against the Homo Sapiens databank (UniProtKB/Swiss-Prot protein knowledgebase released in 2021_4). The signal to Noise (S/N) threshold was set to 1.5. Trypsin was set as enzyme with a maximum of 2 missed cleavage sites; minimum and maximum peptide length was 6 and 144 residues, respectively. Precursor mass tolerance was 10 ppm; fragment mass tolerance was 0.5 Da; use average precursor mass False; use average fragment mass False. Pyruvic Acid (+ 86.000 Da) and Glutathione (+ 305.068 Da) were set as cysteine dynamic modifications. Peptides identification was validated by the Percolator node, with the strict target value of False Discovery Rate (FDR) set at 0.01 and the relaxed value at 0.05. Complete identification of HPLC–MS data of rLF samples trypsin digests carried out at different times of incubation with GSSG (namely, samples rLF GSSG 0 min, rLF GSSG 2 min, rLF GSSG 5 min, rLF GSSG 10 min) are reported in Supplementary Material S1. The data were filtered for high confidence identification and peptide rank 1, according to the Human Proteome Project (HPP) recommendations^39^. Supplementary Material S2 shows the sequence coverage obtained for rLF in samples A) rLF GSSG, 0 min, B) rLF GSSG, 2 min, C) rLF GSSG 5 min and D) rLF GSSG 10, with different colors referring to the confidence level of identification based on False Discovery Rate (FDR) validation by percolator node. Label free relative quantitation of the Lactoferrin identified peptides containing Cysteine in the sequence was assessed by comparing the peak area values (signal/noise ratio > 5) of the eXtracted Ion Current (XIC) plots (average peak area values of three analytical replicates), obtained by extraction of the ion current signals of the relative multiple charged ions (*m/z*) from the total ion current (TIC) profile.

Crystal structure of native LF is derived by PDB id: 1LFG^25^, the structure was drawn using UCSF Chimera^26^. The experimental data reported in figure and tables were analyzed and expressed as Mean ± Standard Deviation (SD). Data were obtained from three independent experiments performed in different days by the operators using the same instruments. The propagation of uncertainties for the quotients ‘enhanced reactivity’ were analyzed according to the classical statistical methods^21^. The graphic and results visualization were obtained using GraphPad Prism software v9.5 (La Jolla, CA, USA).

### Supplementary Information

Supplementary Information 1.

Supplementary Information 2.

## Data Availability

The datasets used and/or analyzed during the current study available from the corresponding author on reasonable request.
