# Supplementary material for: The unusual properties of lactoferrin during its nascent phase
Source: Sci Rep. 2023 Aug 29;13:14113. doi: 10.1038/s41598-023-41064-x (PMC10465537; doi:10.1038/s41598-023-41064-x)
Supplement: Supplementary file 2 — Supplementary Information 2. [file 41598_2023_41064_MOESM2_ESM.pdf]

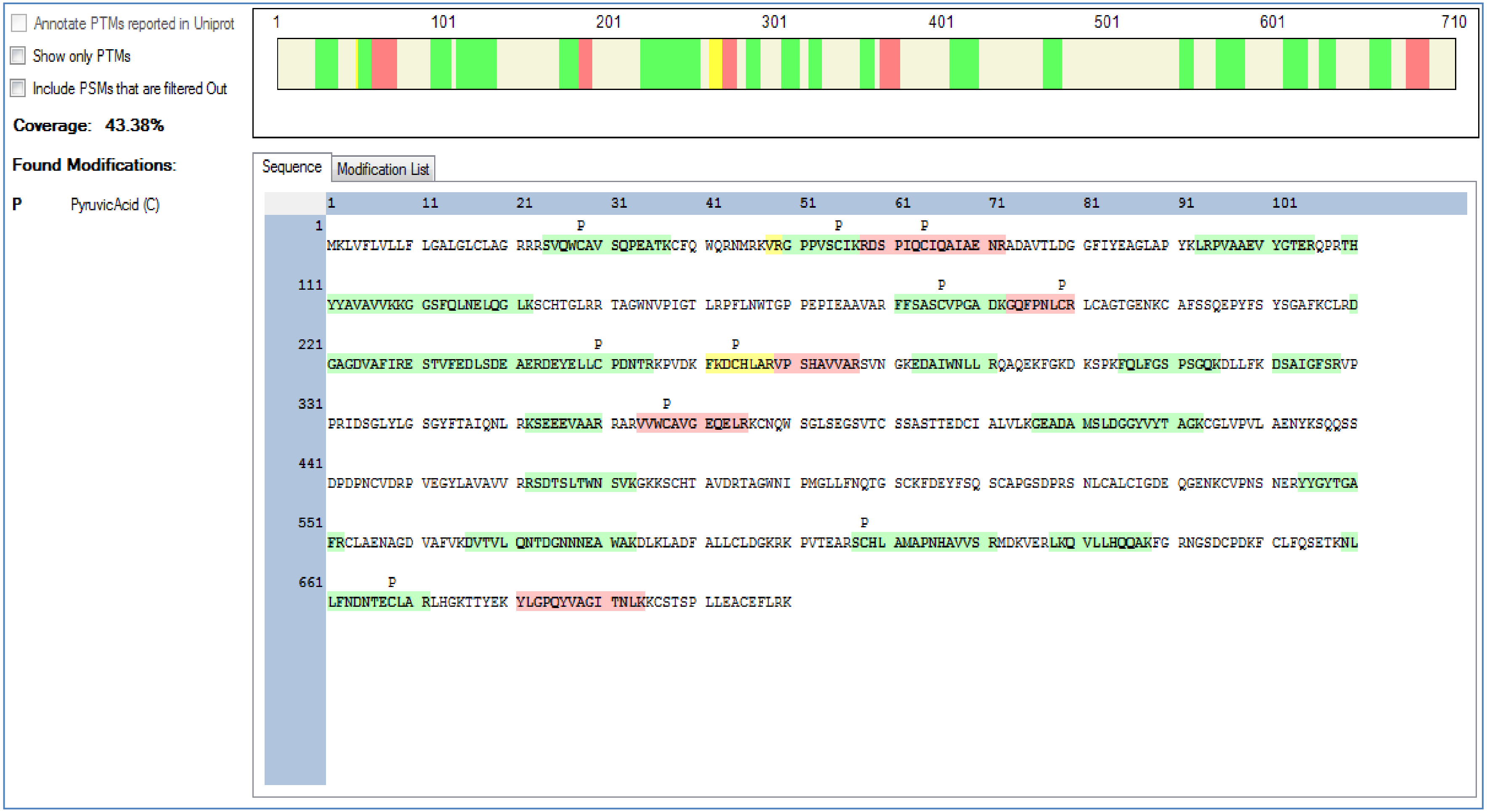

Supplementary Material S2. A) rLF protein identification details in sample **rLF_GSSG_0min**.
The colours indicate the level of confidence of tryptic peptides identification, i.e. green, yellow and pink, for high, medium and low confidence of identication, respectively, based on FDR validation by percolator node.

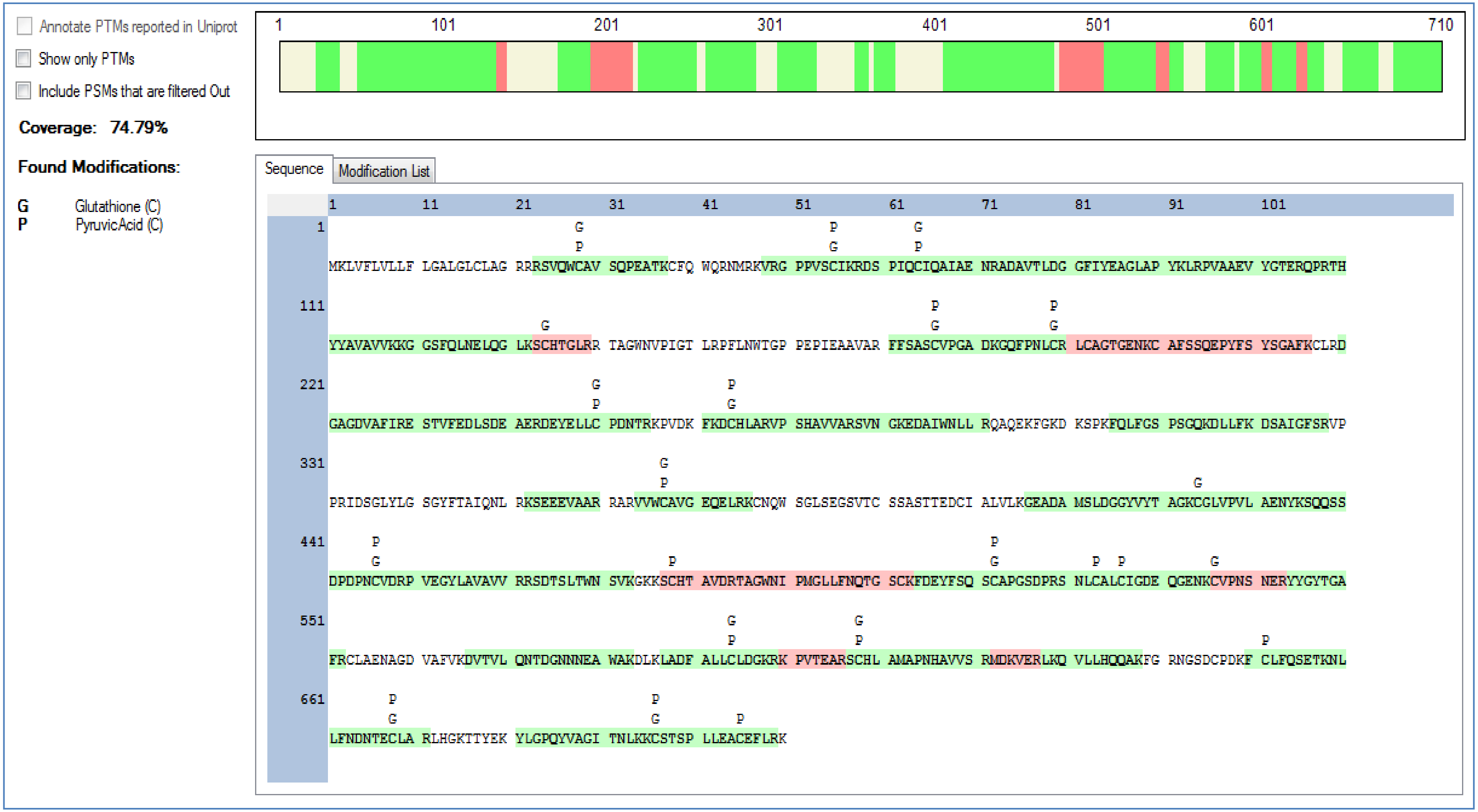

Supplementary Material S2. B) rLF protein identification details in sample **rLF_GSSG_2min**.
The colours indicate the level of confidence of tryptic peptides identification, i.e. green, yellow and pink, for high, medium and low confidence of identication, respectively, based on FDR validation by percolator node.

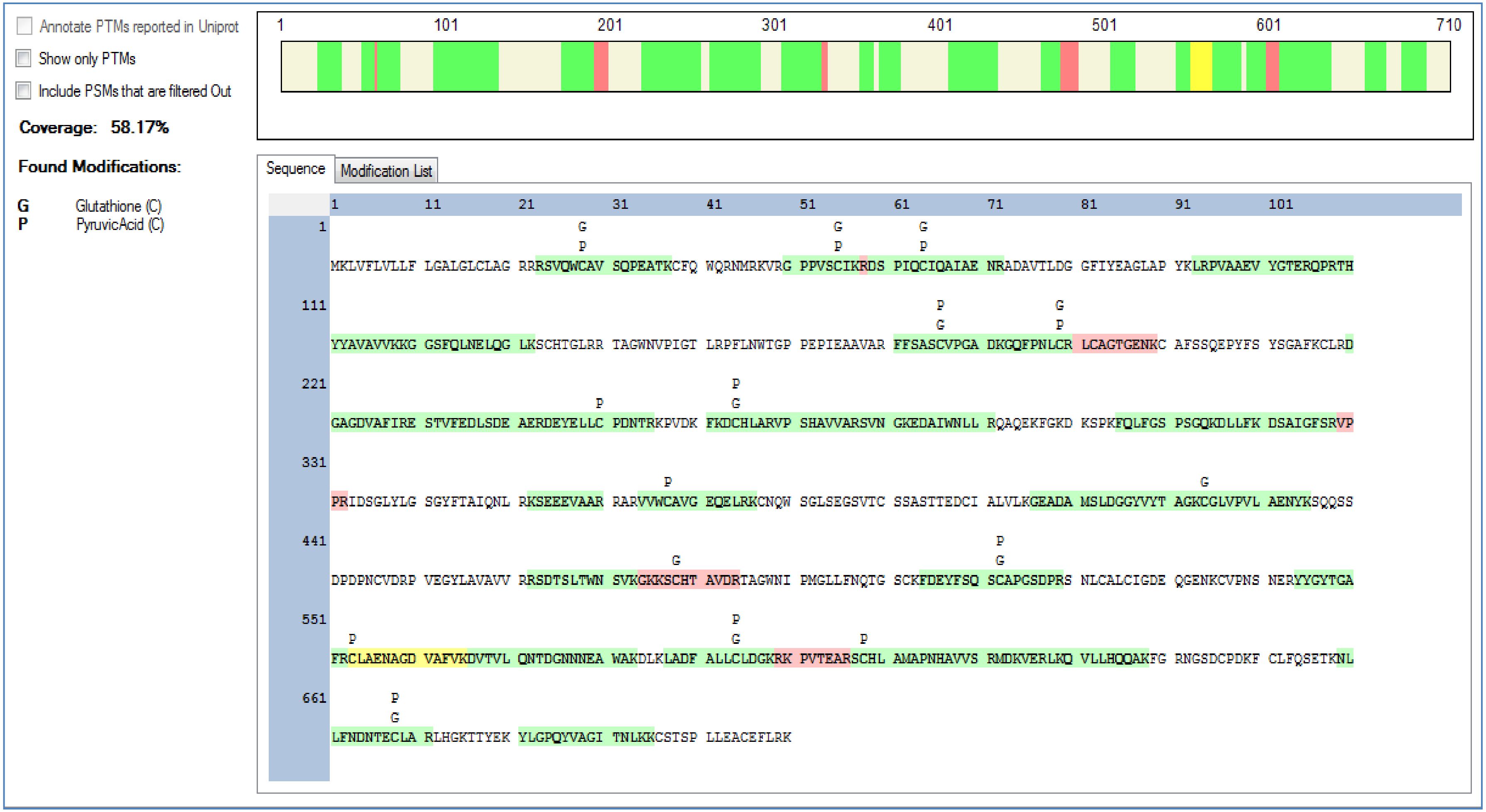

Supplementary Material S2. C) rLF protein identification details in sample **rLF_GSSG_5min**.
The colours indicate the level of confidence of tryptic peptides identification, i.e. green, yellow and pink, for high, medium and low confidence of identication, respectively, based on FDR validation by percolator node.

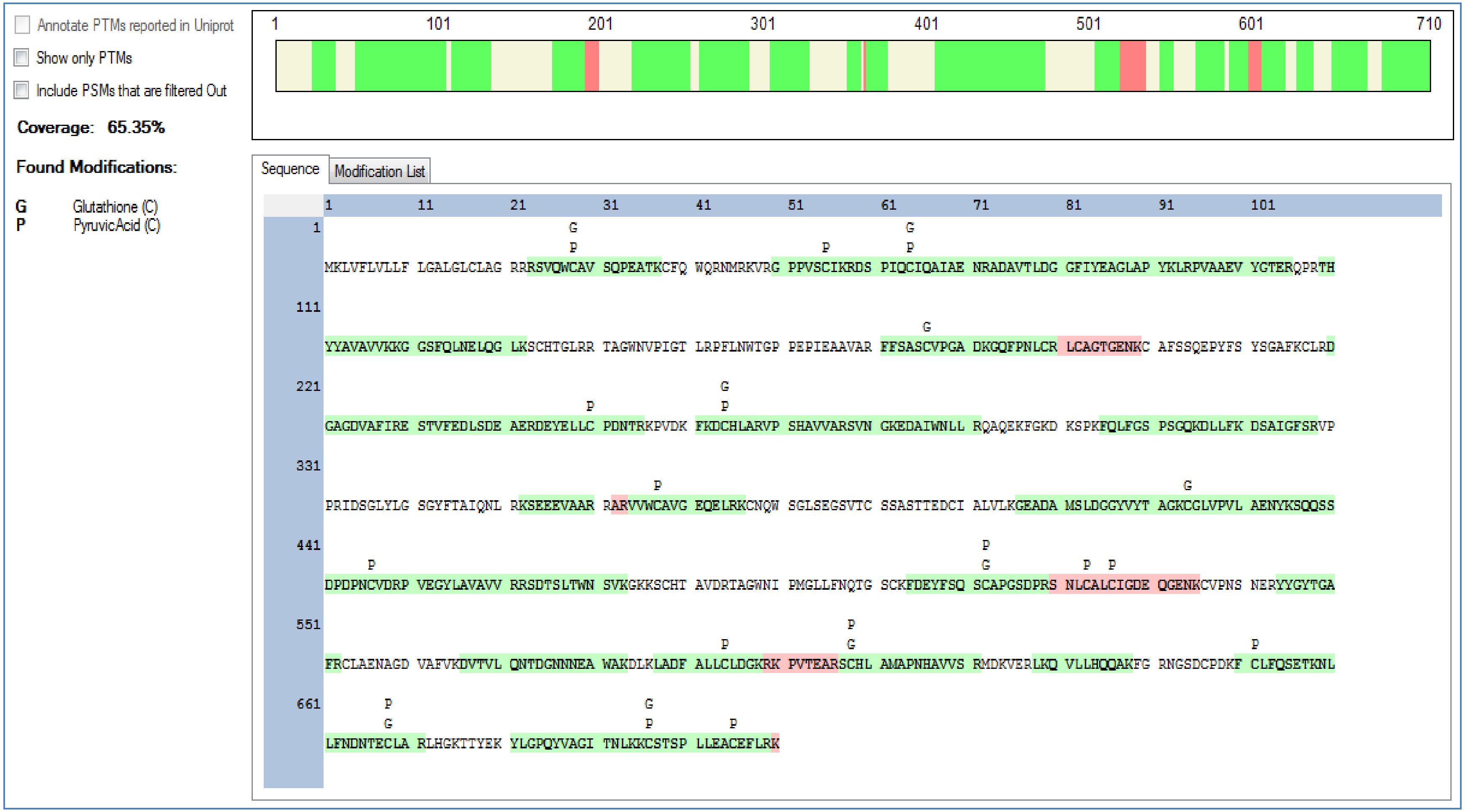

Supplementary Material S2. D) rLF protein identification details in sample **rLF_GSSG_1min**.
The colours indicate the level of confidence of tryptic peptides identification, i.e. green, yellow and pink, for high, medium and low confidence of identication, respectively, based on FDR validation by percolator node.
